# Direct and indirect effects of IFN-α2b in malignancy treatment: not only an archer but also an arrow

**DOI:** 10.1186/s40364-022-00415-y

**Published:** 2022-09-14

**Authors:** Fei Xiong, Qi Wang, Guan-hua Wu, Wen-zheng Liu, Bing Wang, Yong-jun Chen

**Affiliations:** Department of Biliary-pancreatic Surgery, Tongji Hospital, Tongji Medical College, Huazhong University of Science and Technology, Wuhan, Hubei Province China

**Keywords:** Interferon, IFN-α2b, Antitumour effects, Immune response, JAK-STAT pathway

## Abstract

Interferon-α2b (IFN-α2b) is a highly active cytokine that belongs to the interferon-α (IFN-α) family. IFN-α2b has beneficial antiviral, antitumour, antiparasitic and immunomodulatory activities. Direct and indirect antiproliferative effects of IFN-α2b have been found to occur via multiple pathways, mainly the JAK-STAT pathway, in certain cancers. This article reviews mechanistic studies and clinical trials on IFN-α2b. Potential regulators of the function of IFN-α2b were also reviewed, which could be utilized to relieve the poor response to IFN-α2b. IFN-α2b can function not only by enhancing the systematic immune response but also by directly killing tumour cells. Different parts of JAK-STAT pathway activated by IFN-α2b, such as interferon alpha and beta receptors (IFNARs), Janus kinases (JAKs) and IFN‐stimulated gene factor 3 (ISGF3), might serve as potential target for enhancing the pharmacological action of IFN-α2b. Despite some issues that remain to be solved, based on current evidence, IFN-α2b can inhibit disease progression and improve the survival of patients with certain types of malignant tumours. More efforts should be made to address potential adverse effects and complications.

## Background

Malignant tumour is a major public health problem worldwide and the second leading cause of death in the United States. Despite decreased total morbidity and mortality in recent years, the treatment of malignanttumours still faces great challenges, partly due to the high heterogeneity. Tumours of various tissue origins differ in degree of malignancy, and even neoplasms originating from the same tissue show different chemosensitivity. Additionally, due to the lack of specific early clinical symptoms, cancer patients have to face delayed diagnosis and treatment, resulting in an inevitable decline in the clinical diagnosis rate and cure rate [1–3]. Surgery is an option for cancer patients diagnosed at an early stage. Patients with advanced carcinoma and certain types of neoplastic lesions, e.g., neoplastic haematologic disorders and malignant bone tumours, have chemotherapy and radiotherapy as options. The increase in the clinical application of neoadjuvant chemotherapy, targeted therapy and immunotherapy in recent years has provided more options for patients and clinicians and revealed new directions for cancer research and drug development [4, 5].

Interferon-α2b (IFN-α2b) is a highly active cytokine protein that belongs to the interferon-α (IFN-α) family. It is an important form of IFN-α for clinical chemotherapy and immunotherapy (the other form is IFN-α2a). As a pleiotropic cytokine, IFN-α2b has beneficial antiviral, antitumour, antiparasitic and immunomodulatory activities [6, 7]. The Food and Drug Administration (FDA) has approved IFN-α2b as a treatment for hairy cell leukaemia, renal cell cancer and melanoma. A large number of clinical studies on IFN-α2b as therapy for leukaemia, melanoma, renal cell carcinoma and other diseases have been carried out. The combined use of IFN-α2b and surgical treatment, targeted therapy and traditional chemotherapy obviously improves the tumour cell killing effect and compensate for the defects of these treatments alone [8–12].

In this review, we focus on the antitumour effects of IFN-α2b, especially its direct and indirect effect on tumour cells. The pharmacological action of IFN-α2b, related pathway signallings and potential regulators are discussed. Furthermore, we review the findings of clinical studies of the application of IFN-α2b, especially effective compatibilities. Finally, we describe the current challenges of IFN-α2b therapy, including its administration route, side effects, related preparations and markers to provide recommendations for future clinical research.

### Overview of IFN-α family proteins

**Fig. 1:**
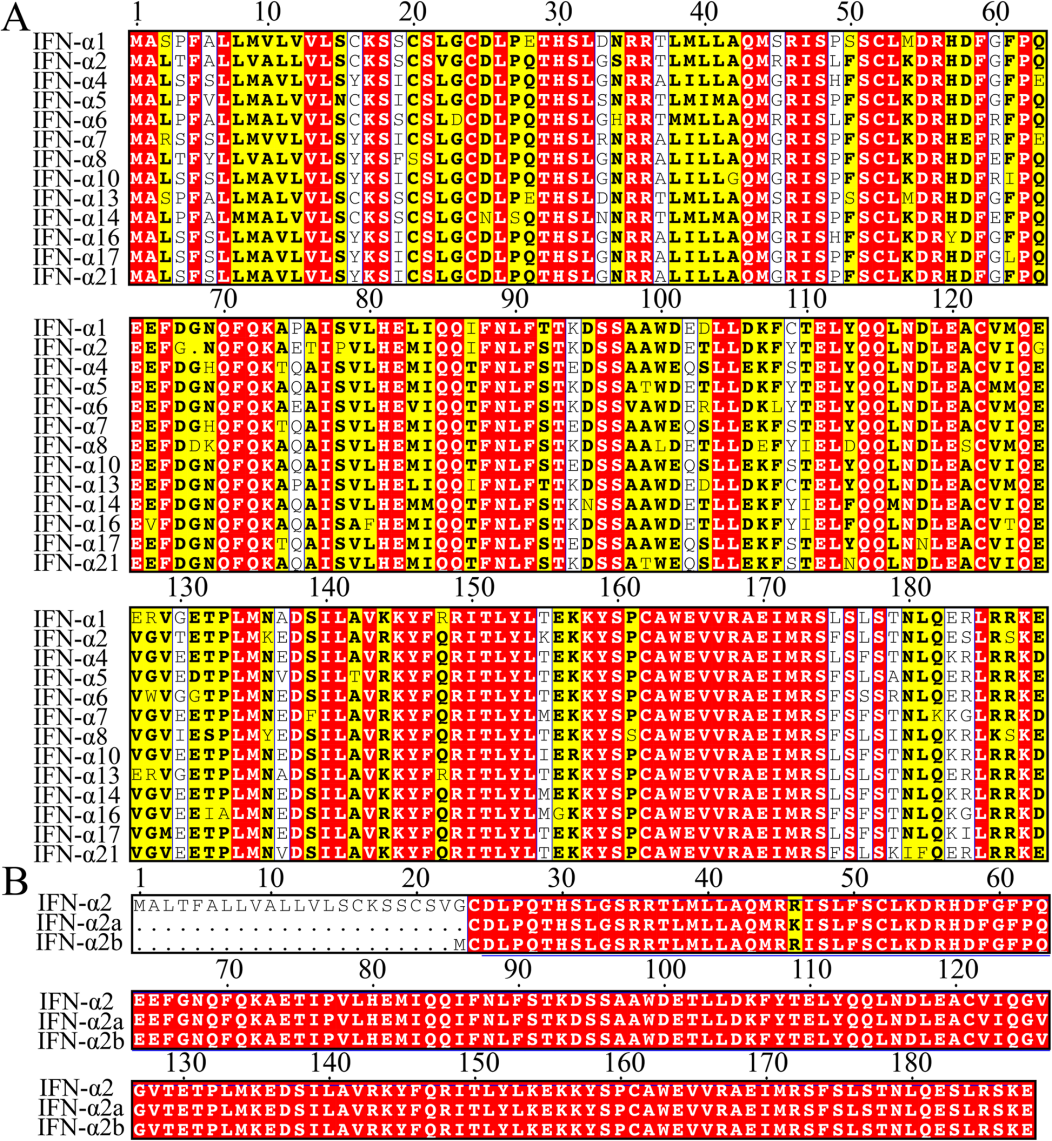
The result of protein sequence alignment. **A** The differences in protein sequences among IFN-α subtypes. **B** The differences in protein sequences among wild-type IFN-α2 and two variants (IFN-α2a and IFN-α2b). The sequences were downloaded from the Protein database (https://www.ncbi.nlm.nih.gov/protein/). Sequence alignment was performed by CLUSTALW and ESPript 3.0. Red box, conservative sequence. Yellow box, high similarity. White box, low similarity

In 1957, Isaacs, A. and Lindenmann, J. first discovered the interferon protein. This protein was named “interferon” because it interferes with the replication of the viral genome [13]. Interferons are divided into three groups, type I (IFN-I), type II (IFN-II) and type III (IFN-III), according to differences in molecular structure and antigenicity. IFN-I family includes IFN-β, IFN-ε, IFN-κ, IFN-ω and at least twenty-four subtypes of IFN-α. IFN-δ derived from pigs and IFN-τ derived from sheep are also listed in the IFN-I family. IFN-I plays an important biological role by binding to the common receptors, interferon alpha and beta receptors (IFNARs) [14]. IFN-α is encoded by fourteen different genes, which could explain the abundance of IFN-α subtypes. The molecular weight of each subtype varies from 16 to 26 kDa, and these subtypes have approximately 70% homologous amino acid sequences (Fig. 1A) [15–18]. Currently, two subtypes of IFN-α2 synthesized by DNA recombination technology, recombinant IFN-α2a and IFN-α2b, are widely used in clinical practice, and they were first tested in phase III clinical studies of certain tumours. Compared with wild-type IFN-α2, the N-terminal of both IFN-α2a and IFN-α2b is deleted. The difference between recombinant IFN-α2a and IFN-α2b is that the 23rd amino acid of IFN-α2a is lysine, while this amino acid is arginine in the IFN-α2b structure (Fig. 1B) [19, 20].

### Antitumour mechanism of IFN-α2b and relevant potential regulatory factors

#### Signalling pathways induced by IFN-α2b: the JAK-STAT pathway

**Fig. 2:**
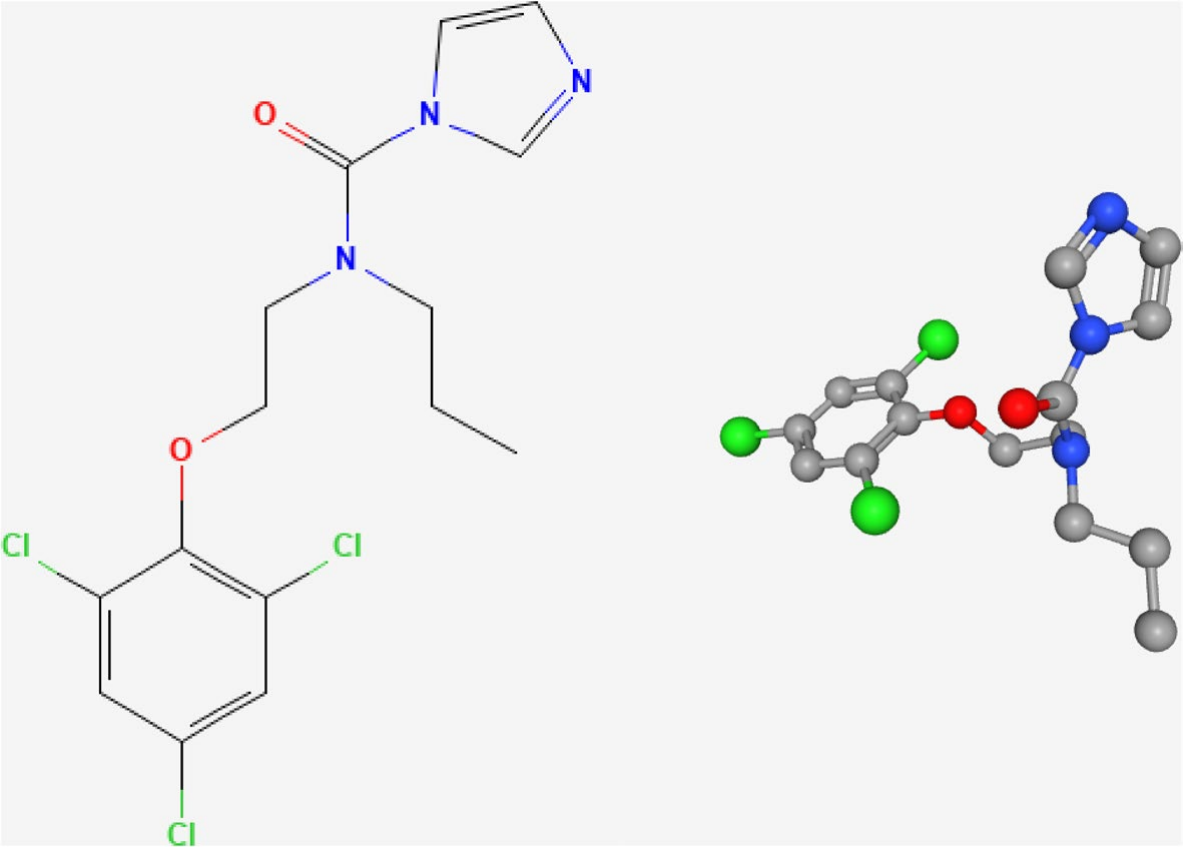
The presentation of the 2D and 3D structures of IFN-α2b. The structures were downloaded from the PubChem database (https://pubchem.ncbi.nlm.nih.gov/)

**Fig. 3:**
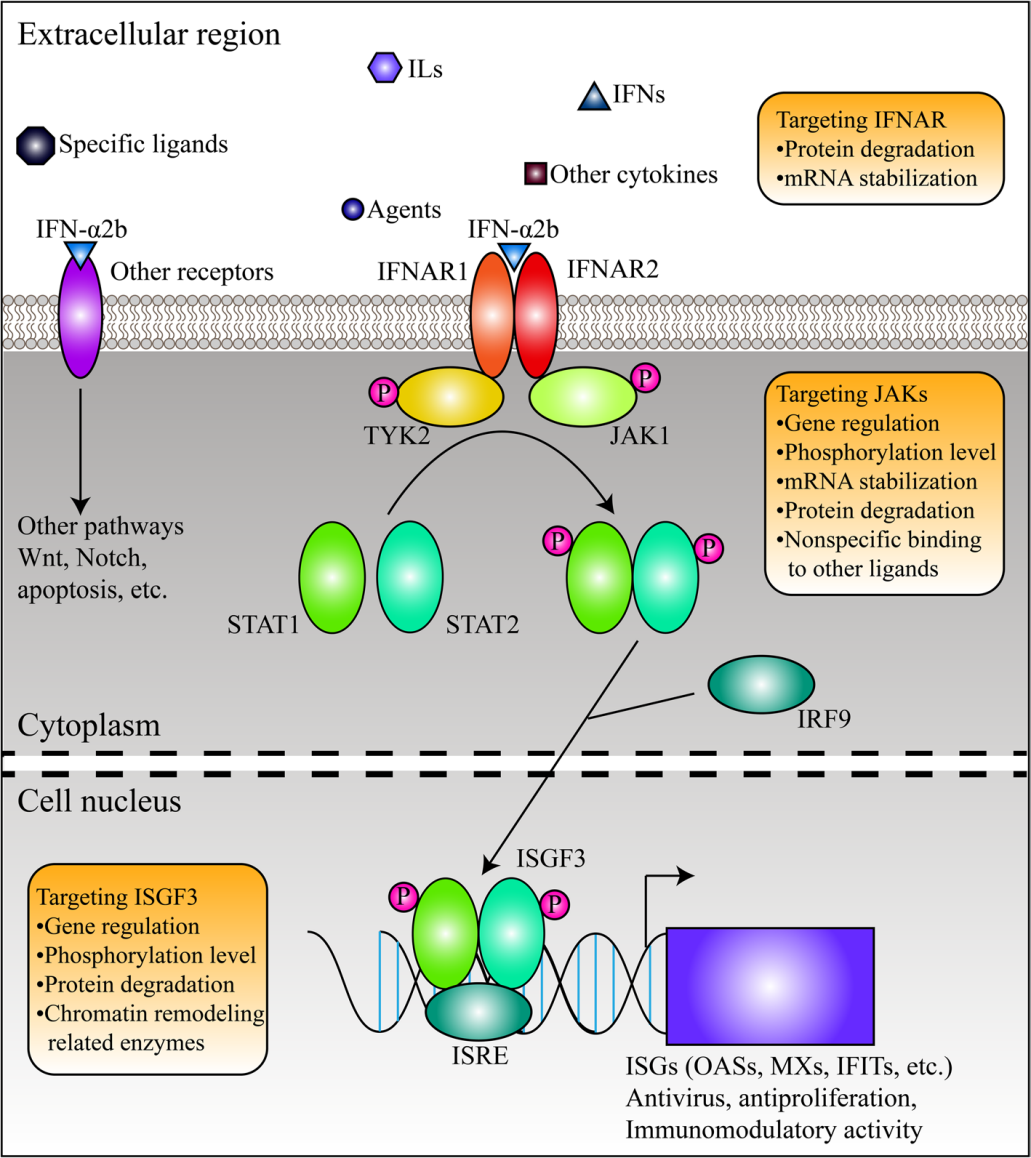
The presentation of the pathways activated by IFN-α2b. The regulatory mechanisms targeting different parts of the pathways are shown in rounded rectangles

The molecular structure of IFN-α2b was shown in Fig. 2. In vivo and in vitro, IFN-α2b can activate the JAK-STAT pathway (also known as IFN-I pathway) [21]. As mentioned above, IFN-α2b can bind to IFNARs. IFNARs are heterodimeric proteins that are ubiquitously expressed and mainly distribute in the cell membrane, and the two domains are encoded by two disparate genes, IFNAR1 and IFNAR2. Once suitable ligands, including IFN-I family members, bind to IFNARs with high affinity, dimerization of IFNAR1 and IFNAR2 is induced, and downstream kinases (also known as Janus kinases, JAKs) are activated, leading to phosphorylation of the substrate, signal transducers and activators of transcription (STATs). Currently, four JAKs (JAK1–3 and tyrosine kinase 2, TYK2) and seven STATs (STAT1–4, 5A, 5B, and 6) have been identified [22]. In the activated IFN-I signalling pathway, IFNAR1 binds to TYK2 and IFNAR2 binds to JAK1 in response to conformational changes of IFNARs (Fig. 3). As a result, JAK1 and TYK2 are activated, and STAT1 and STAT2 are phosphorylated [23, 24]. Then, phosphorylated STAT1 and STAT2 (p-STAT1 and p-STAT2) bind to interferon regulatory factor 9 (IRF9) and form a transcriptional complex named IFN‐stimulated gene factor 3 (ISGF3), which functions by being recruited to conserved genomic regions called the IFN-stimulated response elements (ISREs) and regulates the transcription of downstream IFN‐stimulated genes (ISGs) (Fig. 3) [25–27]. Upregulation of ISGs is one of the signs of IFN-I signalling pathway activation. In the context of certain tumours, autocrine and paracrine activity of IFN-α can amplify interferon signalling [28]. However, once the pathway is activated, some negative regulators also come into play to maintain homeostasis and normal cell activities. For example, suppressor of cytokine signalling 1 (SOCS1) can decrease the phosphorylation level of JAK1 and STAT1. Ubiquitin specific peptidase 18 (USP18) interferes with IFN-I signalling by inducing degradation of ISG15 and disrupting IFNAR2-JAK1 binding [29, 30].

#### Other pathways

Previous studies on the antitumour mechanisms of IFN-α2b have not always focused on the traditional JAK-STAT pathway. For example, crosstalk between IFN-α2b and the Wnt/β-catenin signalling pathway has been found. Ceballos et al. pointed out that IFN-α2b represses this pathway by downregulating β-catenin and the FZD7 protein and blocking the interaction between β-catenin and TCF/LEF factors such as TCF4. As a result, IFN-α2b impedes the proliferation of hepatocellular carcinoma (HCC) cells and induces apoptosis [31]. Additionally, Parody et al. identified a new indirect molecular mechanism. In a rat preneoplastic liver model, IFN-α2b induced the disinhibition of FoxO3a activity. FoxO3a competed for nuclear β-catenin with TCF/LEF and inhibited the Wnt/β-catenin signalling pathway [32].

In addition, despite the limited amount of correlational research, IFN-α2b has been found to activate several pathways, such as the Beclin1 pathway and Notch pathway, involved in multiple intracellular events, such as growth arrest and autophagy, and these pathways are summarized in Table 1. However, it remains to be seen whether and how these pathways interact with the JAK-STAT pathway.

**Table 1:** The pathways that could be activated by IFN-α2b

Cell type	Mechanism	Anticancer action	Notes	Citation
Jurkat, SupT1, H9, CEM, U937	Decreasing the phosphorylation level of MEK1/2 and ERK1/2	Inhibition of cell proliferation	Time-dependence, failure to function within a short period, functioning independently of the upstream signal of Ras and Raf-1	[33]
HepG2	Upregulating BECLIN1 and LC3-II	Induction of autophagy	NA	[34]
Kupfer cells, macrophages, liver parenchymal cells	Activating Caspase-3 and inducing the transport of pSMAD2/3 into nucleus	Induction of apoptosis	Validated in animal models	[35]
Liver parenchymal cells	Activating NADPH oxidase complex and inducing the production of reactive oxygen species	Induction of apoptosis	Validated in animal models	[36]
NA	Upregulting p53 and BAX and downregulating BCL-2 and BCL-xL	Induction of apoptosis	Validated in animal models	[37]
HepG2, Huh7	Downregulating HES1, HES7 and NOTCH1	Inhibition of cell proliferation and induction of cell cycle arrest	Using bone marrow mesenchymal stem cells that could express IFN-α2b protein	[38]
NCI-H295R	NA	Inhibition of cell proliferation and induction of cell cycle arrest and apoptosis	The required dose is too large. IFN-β might be a better choice	[39]
RPMI 8226, U266, NCI-H929	Upregulating TRAIL	Induction of apoptosis	Functioning after 72 h. The function could be antagonized by G1P3 within a short period	[40]
KB	Activating PARP-1	Inhibition of cell proliferation and induction of cell cycle arrest and apoptosis	NA	[41]
SK-MEL-3, SK-MEL-28	Upregulating TRAIL	Induction of apoptosis	Hypermethylation of TNFRSF10A gene could impair the function of IFN-α2b	[42]

#### The direct and indirect effects of IFN-α and the function of ISGs in treating malignancy

Upon IFN-α2b treatment, different biological effects can be induced depending on the type of targeted cells, and these effects can be direct or indirect. First, IFN-α2b can directly act on malignant cells and induce cell cycle arrest, apoptosis and angiogenesis inhibition, having a strong impact on tumour initiation and progression. Second, IFN-α2b can stimulate the indirect effects including immunomodulatory effects on the tumour microenvironment, including enhanced proliferation, maturation and antigen presentation of immune cells such as dendritic cells (DCs), macrophages (Mφ) and natural killer (NK) cells, which strengthens the innate and adaptive immunity to causative agents and malignancy [43, 44]. Third, direct and indirect effects can lead to the lysis of malignant cells and the release of exposed tumor antigen and further strength the antigen presentation and indirect effects. Fourth, more IFN-α can be secreted by these immune cells activated by indirect effects to strengthen the direct effects. As a result, both effects can exhibit synergy against cancerous lesions under ideal conditions.

For virus-infected cells, IFN-α2b treatment leads to a drastic increase in the expression of ISGs and initiates a signalling cascade. ISGs include hundreds of genes that have similar functions in protecting the host from virus entry, replication and spread, including the myxovirus resistance protein family (MXs) and 2'-5'-oligoadenylate synthetase family (OASs) [45]. The molecular mechanisms of antiviral activity include the following: 1. inhibiting the transport of viruses between intracellular and extracellular environments; 2. inhibiting the transcription and translation of the viral genome; and 3. inhibiting viral DNA replication. Interestingly, some ISGs seem to have tumour suppressor properties (reviewed in Table 2), which is in line with the anticancer effect of IFN-α2b. For example, interferon-induced protein with tetratricopeptide repeats 2 (IFIT2) can inhibit the invasion and metastasis of gallbladder carcinoma. MX1 protein can induce autophagy of prostate cancer cells [46, 47]. Nonetheless, the effect of some ISGs on tumours is still controversial. Apolipoprotein B mRNA editing enzyme catalytic subunit 3 (APOBEC3) was previously regarded as an oncogene due to its function in leading to somatic mutation in cervical cancer, but Green et al. demonstrated that some subtypes can increase the fragility of the leukaemia cell genome and increase the sensitivity to apoptosis induced by inhibitors [48, 49]. Eukaryotic translation initiation factor 2 alpha kinase 2 (EIF2AK2) plays a dual role in activating the NF-κB signalling pathway and phosphorylating eukaryotic initiation factor 2α (eIF2α). Thus, its exact function is still unknown [50, 51]. Given this, it could be inferred that the anti-proliferation role of IFN-α2b is the result of overall effect of complex interactions between certain ISGs in the context of different tumours, called ISG portrait, so the carcinogenic function of single ISGs might not conflict with the conclusion. Even so, the relationship between IFN treatment and ISG function is worth investigating, and an understanding of this relationship will provide useful information for clinical treatment of malignancy.

**Table 2:** Some ISGs that were demonstrated as tumor suppressors

Gene symbol	Antiviral mechanism	Cancer type	Anticancer action	Citation
IFIT2	Inhibiting viral protein synthesis and interfering with viral replication	Gallbladder carcinoma	Inhibition of cell proliferation and metastasis	[46, 52–54]
		Colon cancer	Inhibition of cell proliferation and induction of cell cycle arrest and apoptosis	
		Gastric cancer	Inhibition of cell proliferation and migration and induction of cell cycle arrest	
MX1	Acting as GTPase and blocking viral genome transcription	Prostate cancer	Induction of apoptosis and autophagy	[47, 55]
SAMHD1	Inducing the degradation of dNTPs and inhibiting the synthesis of viral DNA	Sézary syndrome	Inhibition of cell proliferation and induction of apoptosis	[56–59]
		Chronic lymphocytic leukemia	Induction of cell cycle arrest and apoptosis	
		Colon cancer	Inhibition of DNA replication	
APOBEC3	Inducing viral genome mutations	Acute myeloid leukemia	Increasing genome fragility and enhancing the anticancer effects of other agents	[48, 49, 60]
		Ovarian cancer	Associated with T cell infiltration	
EIF2AK2	Phosphorylating eIF2α and blocking viral mRNA translation	Melanoma	Inhibition of cell proliferation and induction of apoptosis	[50, 61, 62]
		Breast cancer	Inhibition of cell proliferation and enhancing the anticancer effects of other agents	
		Cervical cancer	Induction of apoptosis	
OAS1	Activating RNase L and interfering viral replication	Breast cancer	Inhibition of cell proliferation and metastasis and induction of cell cycle arrest and apoptosis	[63, 64]

##### Potential regulatory factors of IFN-α2b pharmacological action

In this section, we discuss the potential regulatory factors of the IFN-α2b-related pathway and the possibility of improving the local environment for proper activation of the IFN-I pathway. The regulatory mechanism is shown in Fig. 3.

###### IFNAR

**Fig. 4:**
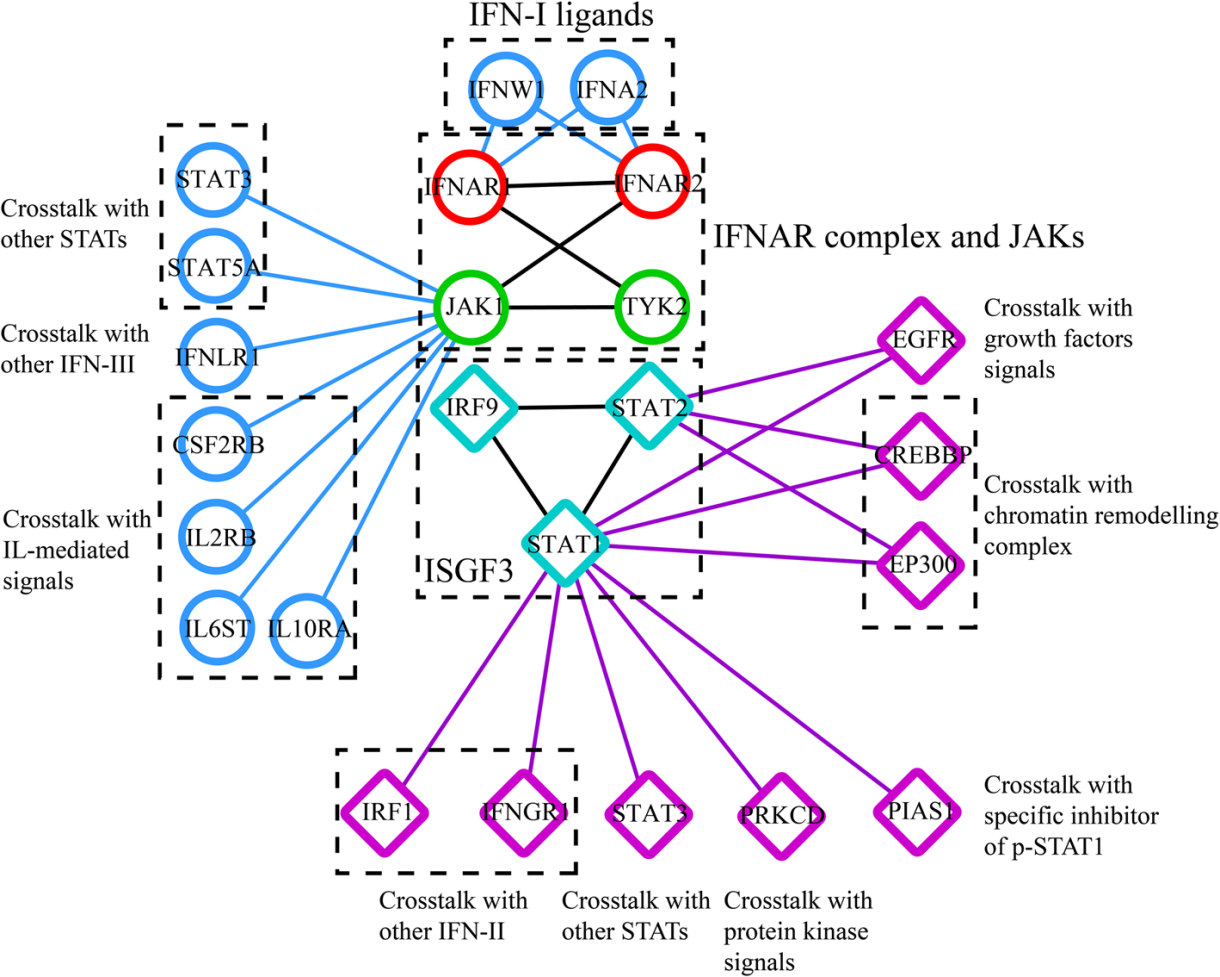
The presentation of the proteins that interacting with different parts of the IFNAR complex, JAKs and ISGF3. The result is retrieved in STRING database (https://cn.string-db.org/) and visualized by Cytoscape. All the proteins are classified by different signalling pathways (black dotted box)

The IFNAR family is composed of two distinct forms, IFNAR1 and IFNAR2, which include two different chains of the receptor for IFN-I and mediate receptor-ligand interactions and transduction of extracellular signals (e.g., IFNA2 and IFNW1) (Fig. 4). Similar to other receptor proteins, IFNARs have extracellular domains (ECDs) and intracellular domains (ICDs). The ECD of IFNAR1 comprises a tandem array of four fibronectin III (FNIII) subdomains, while the ECD of IFNAR2 is characterized by two FNIII-like domains. The IFNAR ICD participates in the interaction between TYK2 and IFNAR1 and between JAK1 and IFNAR2 [65].

In contrast to IFNAR2, IFNAR1 has been a larger focus of mechanistic studies of IFN-I signalling. IFNAR1 is crucial for maintaining an active immune reaction, monitoring abnormal proliferation and maintaining hypersensitivity to IFN-I. Once mutation occurs or deficiency develops, the chances of promoting tumour progression increase. Castiello et al. demonstrated that knockout of IFNAR1 (Ifnar1^−/−^) was associated with earlier onset and marked vascularization in murine breast cancer [66]. The immune-privileged tumour microenvironment induced by IFNAR1 inactivation can be partly explained by interference of immune cell homeostasis. Deletion of IFNAR1 significantly reduces DC-dependent T-cell infiltration into the liver and lung and splenomegaly without altering the frequency of effector/memory-like CD4^+^T cells [67]. Zanker et al. pointed out that the degranulation of NK cells and lysis of breast cancer cells were impaired upon IFNAR1 knockout [68]. Therefore, IFNAR1 could serve as an important mediator of the direct and indirect effect of IFN-I therapy, which has been validated in the context of myeloid-derived suppressor cells and melanoma cells [69, 70].

The regulatory mechanism related to IFNAR1 is shown in Fig. 3. One of the most studied molecular mechanisms is IFNAR1 protein degradation. The IFNAR1 level is strictly controlled by IFNAR1 ubiquitination and degradation facilitated by the SCF-β-TrCP E3 ligase complex, which can bind to phosphorylated sites [71]. PKR-like ER kinase (PERK) regulates the phosphorylation priming process, and subsequently, casein kinase 1 α (CK1α) induces amplification, leading to the recruitment of the E3 ligase complex [72]. Another mechanism is mediated by matrix metalloproteinase-2 (MMP2) in the context of melanoma. MMP2 is secreted from melanoma cells and then cleaves IFNAR1, leading to inactivation of DCs, induction of T helper 2 (Th2) cell polarization and low levels of STAT1 phosphorylation [73]. Both mechanisms could serve as alternative targets for malignancies. Additionally, IFNAR1 can also be regulated at the posttranscriptional level. miR-93-5p targets IFNAR1 and promotes gastric cancer metastasis. miR-208b and miR-499a-5p produced in response to Hepatitis C virus (HCV) infection destabilize IFNAR1 mRNA directly and antagonize IFN-I signalling [74, 75]. In summary, stabilization of IFNAR1 mRNA and blockade of abnormal protein metabolism might be viable strategies to combat the effects of interferon resistance.

###### JAK1 and TYK2

JAK1 and TYK2 belong to the JAK kinase family, a subgroup of the nonreceptor protein tyrosine kinases that possess similar molecular structures. The N-terminal moiety of JAK kinases includes a FERM (band 4.1, ezrin, radixin, moesin) domain and an Src-homology 2 domain (SH2), both of which are structurally important for binding to IFNAR. The C-terminal moiety of JAK constitutes the catalytic kinase domain, whose active conformation can be maintained by phosphorylation of certain tyrosine residues and blocked by an adjacent pseudokinase domain [76, 77]. The catalytic kinase domain exhibits a high degree of homology among the four members. Despite differences in amino acid sequence and molecular structure, JAK3 has 84% sequence homology with JAK1, 87% sequence homology with JAK2 and 80% sequence homology with TYK2 [78].

JAK1 and TYK2 play critical roles in catalysing the phosphorylation process and inducing the active form of STATs to provoke direct and indirect effects. Additionally. JAK1 could bind to IFNLR1, mediating the crosstalk with IFN-III signals (Fig. 4). These two kinases are involved in different pathways, not only the IFN-I pathway but also the JAK1-STAT3 pathway, a harmful carcinogenic signalling pathway in most cases, conflicting with the antitumour effects of JAK1-STAT1 signalling (Fig. 4) [79, 80]. JAKs are necessary for maintaining the numbers and maturation of NK cells, keeping the normal function of Mφ and overcoming immunotherapy resistance, indicating that the role of JAK1-STAT3 pathway in the parenchyma is worth investigating, as the proper immune response is suppressed in cancerous lesions [81–83]. Consistent with this view, JAK1 was found to be critical for inducing resistance to NK cells in melanoma and recurrence of HCC [84, 85]. Indeed, overexpression of both members has been observed in multiple cancer types, along with excessive activation of JAK1-STAT3 signalling. These phenomena may be partly explained by gain-of-function mutations associated with overexpression or hyperactivation. To address this problem, a variety of JAK inhibitors have been developed to block STAT3 signalling by disturbing the phosphorylation-associated activation loop and impairing the enzymatic activity of JAKs. JAK inhibitor (e.g., ruxolitinib and AZD1480) treatment significantly reduced the level of phosphorylated STAT3 and inhibited the proliferation of K-RAS-driven lung adenocarcinoma, small cell lung cancer and triple-negative breast cancer [86–88]. Cirsiliol binds with TYK2 and then suppresses the kinase activity, leading to growth arrest of oesophageal squamous cell carcinoma cells [89]. More exogenic factors are reviewed in Table 3. In the context of hypoxia, JAK1/STAT3 is activated, but transcriptional and translational downregulation of the participants in the IFN-I pathway is also observed. Although targeting JAKs has proven to be a promising strategy, this treatment can interfere with the proper activation of the IFN-I pathway because of the pivotal role of JAKs in the process [43, 90, 91]. The relationship between the two pathways remains to be validated.

**Table 3:** Review of the regulators of JAK1 and TYK2

Regulator	Cell type	Target	Action	Mechanism	Citation
Intracellular factors					
USP6	Mouse preosteoblast cells	JAK1	Protein degradation	Directly binding, leading to deubiquitination and stabilization of JAK1	[92]
TRIM27	HEK293, HeLa	JAK1	Formation of a complex	Directly binding, increasing the binding ability of JAK1	[93]
ALEX1	AGS	JAK1	Functional activation	Increasing the phosphorylation level	[94]
APLNR	HEK293T, A375	JAK1	Formation of a complex	Directly binding	[95]
BRCA1	EcR-293	JAK1	Regulation of gene expression	Transcriptionally upregulating JAK1	[96]
CDK1	A549, 1792	JAK1	Functional activation	Increasing the phosphorylation level	[97]
CISH	Murine NK cells	JAK1	Protein degradation	Directly binding, leading to ubiquitination and degradation of JAK1	[98]
c-KIT	HepG2, SNU398, SNU449	JAK1	Functional activation	Increasing the phosphorylation level	[99]
TJP1	Multiple myeloma cells	JAK1	Functional activation	Decreasing the phosphorylation level in an EGFR-dependent manner	[100]
HIF-1α	Glioma stem-like cells	JAK1	Functional activation	Increasing the phosphorylation level	[90]
SgK223	Pancreatic ductal epithelial cells	JAK1	Functional activation	Increasing the phosphorylation level	[101]
LDL	PC‐3, LNCaP, MIA PaCa‐2, PANC‐1	JAK1	Functional activation	Increasing the phosphorylation level	[102]
MUC1-C	ZR-75–1, MCF-7, MCF-10A	JAK1	Formation of a complex	Directly binding, increasing the binding ability of JAK1	[103]
Annexin A1	EpH4	TYK2	Functional repression	Decreasing the phosphorylation level	[104]
CTLA4	Lymphoma cells and multiple myeloma cells	TYK2	Functional activation	Increasing the phosphorylation level	[105]
MEOX1	MCF-10A, MCF-7, T-47D	TYK2	Regulation of gene expression	Upregulating TYK2	[106]
Exogenic factors					
AH057	HeLa, DU145, HepG2	JAK1	Functional repression	Decreasing the phosphorylation level	[107]
Sorafenib	U87, U251	JAK1	Functional repression	Decreasing the phosphorylation level	[108]
Maraviroc	SUP-B15	JAK1	Functional repression	Decreasing the phosphorylation level	[109]
CDDO-Me	HeLa, MDA-MB-468	JAK1	Functional repression	Decreasing the phosphorylation level	[110]
Myricetin	JB6	JAK1	Functional repression	Decreasing the phosphorylation level	[111]
Leptin	H292, MCF7, MDA-MB-231	JAK1	Functional activation	Increasing the phosphorylation level	[112]
Triptolide	SW480, Caco 2	JAK1	Regulation of gene expression	Downregulating JAK1	[113]
Regorafenib	MEL-RM	JAK1	Functional repression	Decreasing the phosphorylation level	[114]
Oxymatrine	A549	JAK1	Functional repression	Decreasing the phosphorylation level	[115]
4HPR	UMSCC22B	JAK1	Regulation of gene expression	Upregulating JAK1	[116]
HO-3867	Ovarian cancer cells	JAK1	Functional repression	Decreasing the phosphorylation level	[117]
Fraxinellone	A549	JAK1	Functional repression	Decreasing the phosphorylation level	[118]
Formononetin	U266, RPMI 8226	JAK1	Functional repression	Decreasing the phosphorylation level	[119]
Cirsiliol	Esophageal cancer cells	TYK2	Formation of a complex	Directly binding	[89]
PU-H71	MDA-MB-231	TYK2	Regulation of gene expression	Downregulating TYK2	[120]
17-Hydroxy-jolkinolide B	HepG2	TYK2	Regulation of gene expression	Downregulating TYK2	[121]

In addition to inhibitors that are externally administered, JAKs can also be regulated by intracellular proteins in two main ways: 1. modifying the expression of JAKs; and 2. decreasing JAK phosphorylation levels (Fig. 3). First, the protein level of JAKs can be modulated by the degradation pathway. Cytokine-inducible SH2-containing protein (CIS) was found to interact with JAK1, induce proteasomal degradation in NK cells and then mediate resistance to melanoma and prostate and breast cancer metastasis [98]. Quick et al. demonstrated that JAK1 was a substrate for USP6, which leads to deubiquitination of JAK1 and amplification of STAT3 signal [92]. Second, phosphorylation of JAKs can be altered. For example, cytotoxic T lymphocyte-associated antigen 4 (CTLA4) can activate TYK2 by subsequent phosphorylation, resulting in the promotion of lymphoma cell proliferation [105]. Hypoxia inducible factor 1α (HIF-1α) has a similar cellular function to increase the phosphorylation level of JAK1 and promotes the initiation, progression, and recurrence of gliomas [90]. Third, similar to IFNAR1, noncoding RNA (ncRNA) can alter the stability of JAK mRNAs, for example, miR-3677-5p in liver cells, circRNA-9119 in HCC cells and miR-93 in breast stem cells [122, 123]. Similar intracellular factors are reviewed in Table 3, and despite the absence of sufficient evidence, these factors could have the potential to overcome resistance to interferon-based drugs, e.g., BRCA1 (BRCA1 DNA repair associated), a famous target for cancer therapy, significantly upregulates JAK1 expression and interacts with STAT1 dimers, which govern the IFN-dependent anti-proliferative response of breast cancer [96].

One of the reasons for the functional diversity of JAKs might be the nonspecific binding of JAKs and the abundance of ligands. As shown in Fig. 4, JAK1 can interact with multiple receptor proteins, such as CSF2RB, the receptor for IL-3 and IL-5; IL2RB, the receptor for IL-2; IL6ST, the receptor for IL-6 and IL10RA, the receptor for IL-10, indicating that competitive binding of JAKs might give rise to low efficacy of IFN-α2b. After all, most of these ligands have been reported to be principal culprits for abnormal JAK-STAT3 signalling and factors contributing to tumourigenesis. One of the most studied cytokine proteins is leukaemia inhibitory factor (LIF). LIF significantly upregulates the phosphorylation of JAK1 and sustains JAK1/STAT3 signalling by binding to the receptor LIFR, strongly preventing the differentiation of HCC cells and promoting the metastasis and an immunosuppressive phenotype of prostate cancer [124–126]. Targeting such cytokines or cytokine-associated signals might be an effective strategy to address the binding of JAKs (Fig. 3). For example, arsenic trioxide treatment reduces the expression of LIF in HCC [125]. Albrengues et al. found that LIF-induced constitutive phosphorylation of JAK1 was correlated with DNA methylation, which is a well-known, effective target for treating malignancy [124]. Previously, IL-6, IL-11, IL-15 IL-20, and IL-22 were reported to have similar effects on JAK1. IL-6 also contributes to phosphorylation of TYK2 [127–132]. However, in prostate cancer, IL-6 enhances the antiproliferative effect of IFN-α [133]. The exact relationship between interferons and these cytokines depends on the cellular context and needs to be further studied.

In conclusion, potential solutions for overcoming poor response to IFN-α2b include the following: 1. moderate regulation of JAK activity and drug compatibility; 2. targeting of intracellular JAK regulatory factors; and 3. the JAK by targeting harmful ligands and reversing the direction of signalling.

###### ISGF3

ISGF3 is composed of p-STAT1, p-STAT2 and IRF9. STAT1 and STAT2 belong to the STAT family which encodes seven transcription factor proteins characterized by structurally and functionally conserved regions, including the N-terminal domain (NTD), coiled-coil domain (CCD), DNA-binding domain (DBD), linker domain (LD), SH2, tyrosine-phosphorylation (PY) site, and transcriptional activation domain (TAD) [134]. Among these domains, the SH2 domain is susceptible to many STAT inhibitors and mediates homodimerization and heterodimerization (e.g., the STAT1-STAT2 dimer in the activated IFN-I pathway and STAT1 homodimer in the activated IFN-II pathway) along with the NTD. The transcriptional activity of STATs can be regulated by serine phosphorylation of the TAD and recruitment of additional transcriptional activators [135]. CCD mediates the nuclear localization process by selectively binding to the IRF-association domain (IAD) of IRF9 [136].

As essential messengers and mediators of the IFN-I-associated direct and indirect effects, STAT1 and STAT2 can not only provoke STAT-mediated apoptosis, but also participate in enhancing the immune response by upregulating the amount of cytotoxic T lymphocytes (CTLs) and NK cells, both of which are involved in inflammation as well as oncogenesis [136]. STAT1 can act as a negative regulator of T-cell exhaustion and triggers immune responses in head and neck squamous cell carcinoma [137].

ISGF3 can also be regulated by intracellular molecules via an altered phosphorylation level, directly regulating the expression of key components in the complex. The serine threonine-specific protein kinase C-θ (PKC-θ) can increase STAT1 phosphorylation in NK cells in the context of lymphoma [138]. Adenosine deaminase acting on RNA (ADAR1) was reported to block the transport of ISGF3 into the nucleus by upregulating miRNA-302a and thereby targeting IRF9 and STAT1 in gastric cancer [139]. As mentioned above, BRCA1 can also increase the expression of STAT1 and STAT2, providing the evidence of the crosstalk between BRCA1 and IFN-I signalling [140]. Retinoic acid-inducible gene I (RIG-I), an important participant in innate immunity, contributes to IFN-I production and phosphorylation of STAT1 [141]. More factors are reviewed in Table 4. Interestingly, Lu et al. found that in diffuse large B-cell lymphoma, STAT3 could inhibit the expression of STAT1 and STAT2, and Li et al. demonstrated that RIG-I could also be repressed by STAT3 in melanoma.(141, 152) Both studies provide evidence for the antagonism between JAK-STAT3 and the IFN-I pathway, which could be a theoretical basis for the combination of STAT3 inhibitors and IFN-α2b.

**Table 4:** Review of the regulators of ISGF3

ss	Cell type	Target	Action	Mechanism	Citation
Intracellular factors					
TRIM24	Mouse tissue	STAT1	Regulation of gene expression	Transcriptionally downregulating STAT1	[142]
ADAR1	AGS	STAT1	Regulation of gene expression	Downregulating STAT1 by inducing non-coding RNA	[139]
PKC-θ	NK cells	STAT1	Functional activation	Increasing the phosphorylation level	[138]
RARβ	MCF-7	STAT1	Regulation of gene expression	Transcriptionally upregulating STAT1	[143]
KHSRP	A549	STAT1	Functional activation	Increasing the phosphorylation level	[144]
RNF168	NEC, EC109	STAT1	Protein degradation	Directly binding, leading to deubiquitination and stabilization of STAT1	[145]
PR	T47D	STAT1	Functional repression	Directly binding, decreasing the phosphorylation level of STAT1	[146]
HDAC2	A375	STAT1	Formation of a complex	Directly binding	[147]
REST	SK-MEL-28, MM96	STAT1	Regulation of gene expression	Transcriptionally downregulating STAT1	[148]
BRCA1	T47D	STAT1, STAT2	Regulation of gene expression	Transcriptionally upregulating STAT1 and STAT2	[140]
IFIT3	BEL-7402, SMMC-7721	STAT1, STAT2	Formation of a complex	Directly binding, increasing the binding ability	[149]
FBXW7	Melanoma cells	STAT2	Protein degradation	Directly binding, leading to ubiquitination and degradation of STAT2	[150]
p53	HEK-293 T	IRF9	Regulation of gene expression	Transcriptionally upregulating IRF9	[151]
STAT3	Diffuse large B cell lymphoma cells	ISGF3	Regulation of gene expression	Downregulating STAT1, STAT2 and IRF9	[152]
Exogenic factors					
Quercein	B16	STAT1	Regulation of gene expression	Downregulating STAT1	[153]
Dexamethasone	HepG2	STAT1	Functional activation	Increasing the methylation level	[154]
Doxorubicin	MDA-MB 435	STAT1	Functional activation	Increasing the phosphorylation level	[155]
Sodium butyrate	PLC/PRF/5	STAT1	Regulation of gene expression	Upregulating STAT1	[156]
Bortezomib	CNE1, CNE2	STAT1	Functional repression	Decreasing the phosphorylation level	[157]

The function of ISGF3 depends on proper corepressors and coactivators in transcription initiation complexes (TICs). As shown in Fig. 4, protein kinase C-δ (PKC-δ, encoded by PRKCD) influences the phosphorylation level of STAT1 and controls the activation of ISGF3, similar to PKC-θ [138, 158]. Yang et al. found that IFIT3, a known ISG, can bind to STAT1 and STAT2 and enhance the formation of STAT1-STAT2 heterodimers during IFN-α treatment in HCC, indicating the feedback loop between IFN-I signalling pathway and ISGs [149]. Other important factors include IRF1 and IFNGR1, indispensable participants in the IFN-II pathway; EGFR, the receptor of epidermal growth factor (EGF); PR, the receptor of progesterone and PIAS1, a specific inhibitor of p-STAT1 [146, 159–161]. It is worth noting that some histone acetyltransferases, such as EP300 and CREBBP, are also involved (Fig. 4), indicating the correlation between ISGF3 and epigenetic modification mediated by the chromatin remodelling complex. Indeed, Testoni et al. pointed out that upon IFN-α treatment in HCC, ISGF3 was recruited to the promoter of DNp73, an antiapoptotic protein and p53 repressor, along with histone deacetylases (HDACs) and EZH2, a component of polycomb repressive complex 2 (PRC2) [162]. HDACs can be further regulated by S-nitrosylation, as reported in melanoma [147]. In summary, in addition to differences in functional status and intracellular factors, individual differences in patients, which might be evaluated based on the level of endocrine factors, should also be considered. Additionally, targeting epigenetic modifications with inhibitors of downstream regulatory enzymes as an adjunct strategy to IFN-α2b treatment might be an optional worth studying (Fig. 3).

### Clinical application of IFN-α2b in treating malignancy

Owing to its broad-spectrum antiviral roles, IFN-α2b has become an essential part of standard treatments for virus infection. IFN-α2b has been proven to be efficacious in relieving West Nile virus, influenza A virus (IAV) and severe acute respiratory syndrome coronavirus 2 (SARS-CoV-2) infection [163–166]. Moreover, application of IFN-α2b in patients with some virus-related malignancies, such as HIV (human immunodeficiency virus)-related Kaposi sarcoma and HCV-related HCC, was shown to be successful [167, 168]. Progress in treating malignancies not related to virus infection has also been made.

#### Melanoma

The majority of clinical trials involving the application of IFN-α2b as adjuvant treatment for malignancy focus on melanoma. The regimens have included high-dose IFN-α2b (HDI) and low-dose IFN-α2b (LDI). LDI had limited effects in prolonging survival time and lowering potential toxicity. No obvious synergistic therapeutic effect was seen when LDI was combined with other medicines, e.g., bevacizumab [169, 170]. In contrast, the HDI regimen pioneered by Kirkwood et al. was proven to improve the prognosis of patients with high-risk melanoma or ulcerated melanoma [171]. The most utilized protocol is a combination of initial induction therapy (20 MU/m2 i.v. daily for 5 days each week for 4 weeks) and maintenance therapy (10 MU/m2 s.c. three times a week for 48 weeks) [172]. The adjuvant HDI regimen proved effective in prolonging the relapse-free survival (RFS) and overall survival (OS) of melanoma patients in some previous clinical trials, e.g., the E1684 trial in 1996 (RFS: 1 vs. 1.7 years, OS: 2.8 vs. 3.8 years, continuous disease-free rate: 26% vs. 37%) [171]. When the criterion of recruiting melanoma patients was updated in the E1690 trial in 2000 (e.g., patients with T4 primary tumours were included), significant improvement in RFS was still observed, but a difference in OS was not obvious, partly because of the better clinical support and OS improvement of the control group [173]. A meta-analysis combined four randomized clinical trials with large-scale cohorts and found that patients with resected high-risk melanoma could derive RFS and melanoma-specific survival (MSS) benefits from adjuvant HDI [174]. Although the value of HDI has been shown, the results might depend on stage and/or treatment type. IFN-α2b failed to strengthen the immune responses of patients with stage IV melanoma based on the results of enzyme-linked immunospot analysis [175].

To reduce toxicity and improve tolerance, many attempts have been made to design new regimens in combination with IFN-α2b as adjuvant chemotherapy for patients with unresectable melanoma. Unfortunately, the result has not been satisfactory. Among the recently used monoclonal antibody preparations, ecromeximab and ipilimumab proved to be safe in combination with the HDI regimen, whereas no significant improvement in OS was observed [176–178]. The addition of anticancer cytokine preparations, e.g., IL-2, granulocyte-monocyte colony stimulating factor (GM-CSF) and endostatin, failed to induce the expected amplification of the immune response and increased the incidence of adverse reactions in metastatic melanoma patients [174, 179, 180]. Some putative therapeutic regimens composed of traditional chemotherapeutic drugs were found ineffective in clinical trials, e.g., the IFN-α2b + dacarbazine + cisplatin + fotemustine regimen and the IFN-α2b + dacarbazine + cisplatin + carmustine + tamoxifen regimen [181, 182]. Encouragingly, temozolomide, an alkylating agent, was shown to be beneficial for controlling multiple metastases in combination with IFN-α2b. This regimen was demonstrated to be relatively simple, safe and effective and even allows outpatient or home application [183, 184]. Even so, it could be speculated that IFN-α2b is unsuitable for the treatment of metastatic melanoma, partly due to severely impaired immune responses and blocked indirect effects.

#### Haematological tumours

The application of IFN-α2b in leukaemia mainly focuses on the treatment of chronic myelogenous leukaemia (CML). In addition to direct antitumour mechanisms, IFN-α2b was shown to eliminate CML cells by activating circulating PR1-specific CTLs, a subtype of T cells involved in cytogenetic remission and usually deleted in untreated CML [185]. IFN-α2b plus a low-dose cytarabine regimen was used to control chronic-phase myeloproliferation and delay progression to the accelerated phase. After application of the combined regimen, according to Lindauer et al., the survival rate at 3 years was 77%. Maloisel et al. reported a 3-year survival rate of 79% [186, 187]. However, at present, due to its toxicity and unclear application utility, the IFN-α2b regimen is considered a second-line chemotherapy strategy or alternative choice for maintenance therapy. Clinical trials with larger cohorts are needed. Even so, the combination of IFN-α2b with first-line targeted drugs, e.g., dasatinib and imatinib, was demonstrated to induce increased cytogenetic response rates and manageable toxicity [188, 189].

Defective immune surveillance as a result of Epstein–Barr virus (EBV) infection is an important inducer of proliferative disorders and even lymphoma, necessitating the application of IFN-α2b. IFN-α2b can be utilized for low-grade lymphomatoid granulomatosis (LYG), a rare nonmalignant lymphoproliferative disorder, and potentially prevent progression to overt lymphoma by augmenting the immune response to EBV. However, this regimen is not applicable for high-grade lesions that are characterized by insensitivity to immunopotentiators [190, 191]. For the treatment of follicular lymphoma, a kind of indolent B-cell lymphoma, the combination of IFN-α2b with traditional chemotherapeutic drugs was proven to induce a good response, such as the cyclophosphamide + vincristine + prednisone (CVP) + IFN-α2b regimen followed by IFN-α2b maintenance therapy [192, 193]. IFN-α2b therapeutic schedules can also be used for T-cell lymphomas. Geskin et al. argued that IFN-α2b can impair the abnormal immunosuppression mediated by T regulatory cells (Tregs) and myeloid-derived suppressor cells (MDSCs) in Sézary syndrome patients, providing evidence for the medical compatibility of the above strategy [194]. For early-stage Sézary syndrome patients, the psoralen + ultraviolet A irradiation (PUVA) + IFN-α2b regimen is a common systemic combination therapy. The statistical results from three distinct centres indicated complete remission rates of 36%, 73% and 84% [195–197].

#### Digestive system tumours

Strong antiproliferation effects of IFN-α2b were observed in HCC cell lines and an HCC animal model, providing preclinical evidence for the next stage of clinical trials [198]. The combination of IFN-α2b and 5-fluorouracil (5-FU) was suitable for patients with advanced HCC [199]. For HCC patients who had already received radiofrequency ablation, long-term maintenance therapy with IFN-α2b was shown to remarkably inhibit recurrence and prolong survival time [200]. Notably, hepatitis viruses participate in not only the transformation of normal liver to cirrhotic liver, a precancerous lesion, but also further development into HCC. Even when HCC develops, patients can still benefit from antiviral treatment for the prevention of secondary systemic infection and hepatic insufficiency [201]. Hence, the application of IFN-α2b was extended to cases of chronic virus infection, in which it can function to suppress deterioration as a promising complementary therapy for antiviral treatment. The evidence is as follows. First, antiviral drugs, such as telbivudine and entecavir, were able to impair the proliferation of virus-infected HCC cells [202]. Second, the combination of IFN-α2b and antiviral drugs appeared to be more effective than monotherapies. Francesco et al. found that virus infection-related cirrhosis patients with a sustained response to IFN-α2b plus ribavirin tended to have a lower HCC incidence. This regimen overcame the disadvantage of IFN-α2b monotherapy, which failed to improve the clinical outcome, although a curative biochemical response was observed [167, 203, 204]. Third, for those with active hepatitis or cirrhosis, IFN-α2b might prevent the aggravation induced by other risk factors. Liu et al. found that IFN-α2b could significantly relieve ethanol-triggered HBV replication and liver damage [205].

Compared with HCC, gastrointestinal cancer has insufficient clinical data, partly due to poor response and unnecessary side effects. The IFN-α2b + 5-FU + leucovorin regimen showed limited clinical benefits and increased toxicity in patients with rectal carcinoma [206]. In gastric cancer, IFN-α2b administered in a complex regimen and via special delivery methods exerted therapeutic effects [207]. The role of IFN-α2b in the treatment of cholangiocarcinoma is controversial. Kasai et al. argued that the IFN-α2b plus 5-FU regimen was suitable for the treatment of advanced cholangiocarcinoma. Patt et al. added two agents, cisplatin and doxorubicin, to this regimen, yet the curative effects could not counteract the increase in toxicity. To date, a standard regimen containing IFN-α2b has not been determined [208, 209].

#### Other tumours

The application of IFN-α2b is relatively rare in other common cancers. IFN-α2b plus lanreotide was shown to be effective for relieving clinical symptoms and decreasing serum calcitonin levels in advanced medullary thyroid carcinoma patients [210]. The IFN-α2b + all-trans retinoic acid (ATRA) regimen, IFN-α2b + paclitaxel + concomitant radiation regimen and IFN-α2b + cisplatin + 5-FU + leucovorin + concomitant radiation regimen were proven to be effective in advanced renal cell carcinoma, advanced ovarian cancer and advanced nasopharyngeal cancer, respectively [211–215]. In some rare malignancies, such as cutaneous angiosarcoma and Langerhans cell sarcoma, the antitumour effect of IFN-α2b has been validated [216, 217].

Notably, instead of systematic therapy, topical delivery of IFN-α2b has been attempted in certain types of tumours whose primary sites are easy to access, especially ophthalmic neoplasms. Similar topical regimens, such as intravesical delivery of IFN-α2b plus Bacillus Calmete-Guerin (BCG), have been tried in patients with superficial bladder cancer, yet an actual curative effect is controversial [218]. Application of IFN-α2b in eye drops was shown to be successful in the treatment of ocular surface squamous neoplasia and conjunctival epithelial neoplasia [219–222]. For those with refractory epithelial neoplasia, addition of mitomycin C should be considered [221]. However, the decision to apply topical IFN-α2b should be made carefully after the neoplasm is diagnosed to be highly invasive. Although no significant differences in the recurrence rate between surgery and IFN-α2b were found by Nanji et al., the reduction of cancer cell burden induced by surgery was not achieved by IFN-α2b [222]. Selection of either treatment should be based on pathologic stage and grade.

### The current challenges of IFN-α2b treatment

Many studies have shown the antineoplastic role of IFN-α2b, which has been approved by the FDA for the treatment of certain kinds of tumours. However, there are several factors that have prevented IFN-α2b from becoming an established therapy. First, due to tumour heterogeneity and differences in mutational burden, certain types of tumours have relatively poor responses to IFN-α2b treatment at the molecular mechanism level [223]. Second, the reports about alternative biomarkers for the inclusion of the patients suitable for IFN-α2b treatment and indicators for controllable therapeutic responses in long-term IFN-α2b therapy are greatly needed. Third, IFN-α commonly causes almost inevitable side effects in certain cases, probably because of the nonspecific stimulation and enhancement of inflammation pathways and off-target effects on non-neoplastic tissues. Fourth, various IFN-α2b formulations differ in bioavailability and tissue specificity.

#### Challenges in the poor response to IFN-α2b treatment caused by multiple factors

As mentioned above, the proper induction of IFN-I signalling pathway requires to satisfy many conditions, e.g., intact JAK-STAT pathway. However, in cancerous lesions, endogenous IFN-I signals are significantly severely suppressed, which could be partly explained by tumour heterogeneity and interactions among various cell subsets. Additionally, mutational burden arising from hypoxia stress and overgrowth can interrupt the IFN-I pathway, which should be taken seriously. JAK mutations, such as TYK2 mutations in malignant peripheral nerve sheath tumours (approximately 60%) and acute leukaemia (approximately 40%) and JAK1 mutations in acute leukaemia (27.3%) and certain subtypes of prostate cancer (68%) could inhibit the IFN-I signals and even reverse the biological effect [224–227]. Substantial dysfunction of ISGF3 is found as well. In addition to the downregulation of STATs caused by underlying molecular mechanisms, accumulated mutations (e.g., STAT1-Y701F and STAT2-Y690F) also play important roles in preventing ISGF3 formation and thereby blocking IFN signals, indicating that it is necessary to evaluate the functional status of the participants in the pathway before deciding on IFN-α2b treatment [228, 229].

Target therapies via virus vectors, called oncolytic virus therapies, were recently developed and hopeful for overcoming the resistance to IFN-I. Oncolytic virus (OV) refers to these modified virus vectors that can specifically infect cancer cells [230]. The replication of OV is more effective in tumour than normal tissues partly due to unique characteristics of malignant cells, such as abnormal signal activation (e.g., RAS/RAF1/MEK/ERK signalling pathway), overactive biosynthesis function and instable genome for the convenience of viral genetic recombination [231]. The antitumour mechanism of OV was summarized as follows: First, the replication and accumulation of OV can provoke the lysis of malignant cells. Second, the residual lysis and OV itself could stimulate the innate and adaptive immunity by cytokine secretion and antigen presentation. Third, the coding genes carried by genetically engineered OV vectors could facilitate the antitumour effects [232].

The relation between IFN-I and OV remains controversial. As OV derives from wild-type virus with pathogenicity and immunogenicity, the activation of IFN-I signals upon the systematic or intratumoural application of OV (e.g., induction of certain ISG portraits) is regarded as antiviral and protective responses, which limits the normal antitumour oncolytic process. On the contrary, the impact of OV could be maximized in those lesions with minimal T cell infiltrates, absence of IFN-I signatures, and the presence of immunosuppressive cells [233]. The percentage of infected benign prostate cells is lower than metastatic cancer cells under the same multiplicity of infection (MOI) indicating the amplification of OV genomes significantly slows down because of intact IFN-I signalling. In contrast, glioma tumour cells and melanoma cells, characterized by the restricted intrinsic IFN-I responses, show better sensibility to OV and worse cell viability [234–236]. Additionally, OV contributes to the specific pattern of immune cell infiltration (e.g., CD8^+^ T cells and CD4^+^T cells) especially in IFNAR-knockout tumours [237]. When IFN-I signal was blocked by JAK inhibitors, the progeny yield of OV significantly increased, indicating enhanced oncolysis process [238, 239]. Nevertheless, on the other hand, moderate activation of IFN-I response needs to be maintained endogenously or exogenously for the following reasons. First, OV and induced IFN-I responses have common antitumour mechanism, e.g., increasing the phosphorylation level IRF3 for sustained secretion of IFN-I [240]. Second, appropriate levels of IFN-I can sustain IFN-I-dependent stimulation to NK cells, necessitating the insertion of IFN-I or tumour antigen encoding genes into the structure of OV. Notably, excessively high dose of IFN-I may decrease the NK cells in peripheral circulation [230, 241, 242]. Third, in some cases, the cytotoxicity of OV seems to be independent of antitumour IFN-I signals. For example, in the context of pancreatic cancer, OV infection is not influenced by ISG upregulation and the activation of the stimulator of interferon genes (STING). In glioblastoma cells, knockdown of certain ISGs can even inhibit the proper function of OV [242–244]. Recently, the strategy that inhibits the IFN-I signalling during the initial phase of OV infection by small molecule chemical (BLT-1) was proved to be effective to induce efficient virus replication and strong immune response, which balanced both sides of IFN-I functions [245].

#### Challenges and advances in the inclusion of patients and indication of therapeutic effects

Long-term IFN-α2b therapy is often chosen to induce clinical remissions and prevent future recurrence. However, as mentioned above, it is far from a reasonable choice to apply IFN-α2b to those with exhausted immune systems and poor general conditions (e.g., elevation of aminotransferase, renal hypofunction and simultaneous peripheral blood cell reduction). The appropriate application parameters and indications are worth investigating, though these are common problems of all the toxic chemicals for chemotherapy. Except for traditional laboratory diagnosis, diagnostic biomarkers are one of many possible solutions for confirming the necessity and feasibility of IFN-α2b therapy in different patients. Tarhini et al. pointed out that IFN-α2b should be given to patients with a higher mortality risk to avoid unnecessary toxicity and cost. This risk could be evaluated by a prognostic model based on tumour necrosis factor alpha receptor II (TNF-RII), transforming growth factor alpha (TGF-α), tissue inhibitor of metalloproteinases 1 (TIMP-1) and C-reactive protein (CRP) levels [246].

During the IFN-α2b treatment, indicators of curative effects can be helpful for later decisions. IFN-α2b can induce a state of autoimmunity that is beneficial to the prognosis, which can be detected by the measurement of autoantibodies [247]. Some components of the IFN-I pathway, such as p-STAT1 and ISGs, can also indicate the pharmacological response to IFN-α2b [21]. In a joint laboratory and clinical trial (E1690), modulation of intercellular adhesion molecule 1 (ICAM1) was observed by flow cytometry upon IFN-α2b treatment [248]. Additionally, two studies from distinct centres focused on the role of cytokines, which are relatively easy to detect and could be potential markers for monitoring disease progression during IFN-α2b therapy. Yurkovetsky et al. found that upon IFN-α2b treatment, the levels of proinflammatory cytokines, including serum tumour necrosis factor-α (TNF-α) and various ILs, were upregulated, while the levels of angiogenic factors, including vascular endothelial growth factor (VEGF) and EGF, were downregulated [249]. Hofmann et al. found that in stage II and III melanoma patients, a low level of TNF-α indicated a poor response and unfavourable RFS, while a high level of serum TNF-α correlated with toxicity [250]. Despite all the progress noted above, most conclusions have come from observational studies. The precise role of these markers in clinical practice remains unclear and needs to be validated by higher-level evidence.

#### Challenges in the adverse effects and complications triggered by IFN-α2b

One of the important factors limiting the large-scale application of IFN-α2b in treating malignancy is the prevailing adverse effects, although they are not life-threatening in most cases. In a retrospective study of melanoma, 71% of the patients were found to experience at least one kind of adverse effect, but no drug-related death was observed [251]. Another study on Sézary syndrome reported a similar percentage of patients who experienced a toxic response (60%) [196]. The common adverse effects in studies using IFN-α2b monotherapy have been reviewed. The most frequent side effects were fever and fatigue. In addition, adverse effects of the digestive system of different grades were also common, such as anorexia, diarrhoea, vomiting and hepatic dysfunction. Furthermore, haematologic toxicity could also be observed, e.g., neutropenia, thrombocytopenia and anaemia. Neurologic complications, e.g., anxiety, depression and cognitive disturbance, and metabolic complications (e.g., hyperglycaemia and hyperlipemia), were relatively unusual. The most common adverse effect was influenza-like symptoms, featuring a series of symptoms including fever, fatigue, rigors, arthralgia, myalgia, headache and sweating, indicating that this complication might be associated with nonspecific activation of the inflammatory response. Although most adverse effects were found to be moderate, they were undesirable and painful for many patients who received IFN-α2b, leading to a relatively high rate of treatment discontinuation, especially for those with poor overall health caused by primary disease. Most adverse effects could be relieved by dose reduction and even drug withdrawal, which was accompanied by a dose-dependent delay in a strong therapeutic response. In addition, poor compatibility often induced unnecessary complications, and the overall survival was not improved, which are challenges for the application of IFN-α2b in the treatment of multiple tumours [169]. Even so, most side effects can be relieved by symptomatic treatments, e.g., the application of non-steroid anti-inflammatory drugs (NSAIDs) and colony-stimulating factor preparations could relieve fever and leukocytopenia, respectively. Serious adverse effects, such as hypohepatia, proteinuria and myelodysplasia and neuropsychiatric symptoms, can be solved by medication discontinuation and convalescence in most cases.

#### Challenges and advances in the delivery and preparation of IFN-α2b

In clinical use, IFN-α2b is usually administered subcutaneously to exert antitumour effects [206, 211, 252]. Some studies have reported that intravenous administration is beneficial for promoting lymphatic absorption and targeting the lesion site [253]. For the treatment of ophthalmic tumours, subconjunctival injection and eye drops can be considered as well [254]. Additionally, IFN-α2b in combination with a melanoma vaccine can enhance the antigen presentation response to kill tumour cells in the treatment of melanoma. New vaccine preparations, such as HeberFERON, are available but are still controversial [174, 255–257]. In addition, the overexpression of IFN-α2b induced by adeno-associated virus vectors (AAV) can inhibit the proliferation of prostate cancer cells, which has been validated in animal studies [258–261].

Natural IFN-α2b has antiviral, anticancer, antiparasitic and immunoregulatory pharmacological effects. Nonetheless, it has disadvantages as well, such as poor stability and potential immunogenicity, which could lead to mostly uncontrollable biological degradation. For patients with cancer, this degradation necessitates an excessive dose to achieve ideal therapeutic effects, which increases the incidence of complications and limits wide usage [262, 263]. To address this problem, pharmaceutical advances to add poly (ethylene glycol) (PEG) modifications to natural IFN-α2b molecules, named PEG-IFN-α2b, have been made. This preparation maintains the advanced structures of IFN-α2b, inhibits drug metabolism, and decreases filtration by the kidney, resulting in a prolonged plasma half-life and a lower frequency of administration (once per week) [264, 265]. Although the modification decreases the biological activity of IFN-α2b and its binding ability with interferon receptors, there is no significant difference in antiviral and antitumour effects in vitro [266]. In recent years, more IFN-α2b preparations have been developed, such as IFN-α2b with polysarcosine modifications and cyclic chimeric IFN-α2b. However, the clinical efficacy has not been supported by sufficient evidence and needs to be tested in more clinical studies. Moreover, excessive modification of IFN-α2b might result in heterogeneous mixture of isomers and decrease efficiency, indicating that the balance should be achieved by more evidence of pharmacokinetics and clinical trials [267, 268].

## Conclusions and future perspectives

IFN-α2b clearly plays a role in antitumour therapy, not only by enhancing the systematic immune response but also by directly killing tumour cells, just as a role serving as an archer as well as an arrow. Based on the current evidence, IFN-α2b inhibits disease progression in and improves the survival of patients with certain types of malignant tumours, such as melanoma, leukaemia, lymphoma and HCC. In addition, because compared with other antitumour agents, IFN-α2b preparations can be relatively easily and inexpensively delivered, IFN-α2b might be a convenient solution for outpatient medication and therapy in areas with lower economic status and without access to newly developed drugs.

Despite the advantages mentioned above, after reviewing all the literature, we found that most major breakthroughs were made before 2010. In addition, the extension of IFN-α2b treatment to other tumours has been unsatisfactory and beset with challenges. One of the critical reasons is the adverse effects and complications that, as mentioned above, lead to interruption of therapy plans and increase potential biases of clinical trials, such as reporting bias, diagnostic suspicion bias and observer bias. Therefore, addressing the drug resistance and nonspecific inflammatory responses caused by IFN-α2b should be a focus of future studies. According to the majority of available evidence, the following aspects may lead to solutions. First, there should be reasonable strategies for administering further IFN-α2b treatment in patients. The general conditions should be evaluated by strict physical examination and laboratory inspection. Gene detection can be conducted before IFN-α2b treatment to recognize potential alterations of the important participants in the JAK/STAT pathway. Second, evidence-based therapies combining pharmacotherapies with IFN-α2b should be developed, as some of the clinical trials seemed to have few or weak evidences in phase I clinical study. These combinations should be based on the animal studies and mechanism researches associated with IFN-α2b or members of related pathways, e.g., the JAK/STAT pathway, rather than simply on the clinical experience or historical documents. Third, a timely plan for dealing with potential adverse effects and complications of IFN-α2b should be developed, which deserve serious consideration by researchers and operators. Fourth, development of new preparations of IFN-α2b should be encouraged. In particular, agents with higher tissue targeting specificity and higher potency should be developed for advanced-phase clinical trials.

## Data Availability

Not applicable.

## Abbreviations

5-FU: 5-Fluorouracil
AAV: Adeno-associated virus vectors
ADAR1: Adenosine deaminase acting on RNA
APOBEC3: Apolipoprotein B mRNA editing enzyme catalytic subunit 3
ATRA: All-trans retinoic acid
BCG: Bacillus Calmete-Guerin
BRCA1: BRCA1 DNA repair associated
CCD: Coiled-coil domain
CIS: Cytokine-inducible SH2-containing protein
CK1α: Casein kinase 1 α
CML: Chronic myelogenous leukaemia
CRP: C-reactive protein
CTL: Cytotoxic T lymphocyte
CTLA4: Cytotoxic T lymphocyte-associated antigen 4
CVP: Cyclophosphamide + vincristine + prednisone regimen
DBD: DNA-binding domain
DC: Dendritic cell
EB: Epstein–Barr virus
ECD: Extracellular domain
EGF: Epidermal growth factor
EIF2AK2: Eukaryotic translation initiation factor 2 alpha kinase 2
eIF2α: Eukaryotic initiation factor 2α
FDA: The Food and Drug Administration
FNIII: Fibronectin III subdomain
GM-CSF: Granulocyte-monocyte colony stimulating factor
HCC: Hepatocellular carcinoma
HCV: Hepatitis C virus
HDAC: Histone deacetylase
HDI: High-dose IFN-α2b
HIF-1α: Hypoxia-induced hypoxia inducible factor 1α
HIV: Human immunodeficiency virus
IAD: IRF-association domain
IAV: Influenza A virus
ICAM1: Intercellular adhesion molecule 1
ICD: Intracellular domain
IFIT2: Interferon-induced protein with tetratricopeptide repeats 2
IFNAR: Interferon alpha and beta receptor
IFN-α2b: Interferon-α2b
IFN-I, II, III: Type I, II, III interferon
IL: Interleukin
IRF: Interferon regulatory factor
ISG: Interferon‐stimulated gene
ISGF3: Interferon‐stimulated gene factor 3
ISRE: Interferon-stimulated response element
JAK: Janus kinase
LD: Linker domain
LDI: Low-dose IFN-α2b
LIF: Leukaemia inhibitory factor
LYG: Lymphomatoid granulomatosis
MDSC: Myeloid-derived suppressor cell
MMP2: Matrix metalloproteinase-2
MSS: Melanoma-specific survival
MX: Myxovirus resistance protein family
Mφ: Macrophage
ncRNA: Noncoding RNA
NSAID: Non-steroid anti-inflammatory drug
NK: Natural killer cell
NTD: N-terminal domain
OAS: 2'-5'-Oligoadenylate synthetase family
OS: Overall survival
OV: Oncolytic virus
PEG: Poly (ethylene glycol)
PERK: PKR-like ER kinase
PKC: Protein kinase C
PRC2: Polycomb repressive complex 2
PUVA: Psoralen + ultraviolet A irradiation regimen
PY: Tyrosine-phosphorylation site
RFS: Relapse-free survival
RIG-I: Retinoic acid-inducible gene I
SARS-CoV-2: Severe acute respiratory syndrome coronavirus 2
SH2: Src-homology 2 domain
SOCS1: Suppressor of cytokine signalling 1
STAT: Signal transducers and activators of transcription
STING: The stimulator of interferon genes
TAD: Transcriptional activation domain
TGF-α: Transforming growth factor alpha
Th2: T helper 2 cell
TIC: Transcription initiation complex
TIMP-1: Tissue inhibitor of metalloproteinases 1
TNF-RII: Tumour necrosis factor alpha receptor II
TNF-α: Tumour necrosis factor-α
Treg: T regulatory cell
TYK2: Tyrosine kinase 2
USP18: Ubiquitin specific peptidase 18
VEGF: Vascular endothelial growth factor
